# Immunotoxicity Considerations for Next Generation Cancer Nanomedicines

**DOI:** 10.1002/advs.201900133

**Published:** 2019-08-01

**Authors:** Gary Hannon, Joanne Lysaght, Neill J. Liptrott, Adriele Prina‐Mello

**Affiliations:** ^1^ Nanomedicine and Molecular Imaging Group Trinity Translational Medicine Institute (TTMI) Trinity College Dublin Dublin 8 Ireland; ^2^ Department of Surgery TTMI Trinity College Dublin Dublin 8 Ireland; ^3^ Department of Molecular and Clinical Pharmacology Institute of Translational Medicine The University of Liverpool Liverpool L69 3GF UK; ^4^ Laboratory for Biological Characterisation of Advanced Materials (LBCAM) TTMI Trinity College Dublin Dublin 8 Ireland; ^5^ Advanced Materials and Bioengineering Research (AMBER) Centre CRANN Institute Trinity College Dublin Dublin 2 Ireland

**Keywords:** cancer, immunotoxicity, nanomedicine, regulation, safety

## Abstract

Although interest and funding in nanotechnology for oncological applications is thriving, translating these novel therapeutics through the earliest stages of preclinical assessment remains challenging. Upon intravenous administration, nanomaterials interact with constituents of the blood inducing a wide range of associated immunotoxic effects. The literature on the immunological interactions of nanomaterials is vast and complicated. A small change in a particular characteristic of a nanomaterial (e.g., size, shape, or charge) can have a significant effect on its immunological profile in vivo, and poor selection of specific assays for establishing these undesirable effects can overlook this issue until the latest stages of preclinical assessment. This work describes the current literature on unintentional immunological effects associated with promising cancer nanomaterials (liposomes, dendrimers, mesoporous silica, iron oxide, gold, and quantum dots) and puts focus on what is missing in current preclinical evaluations. Opportunities for avoiding or limiting immunotoxicity through efficient preclinical assessment are discussed, with an emphasis placed on current regulatory views and requirements. Careful consideration of these issues will ensure a more efficient preclinical assessment of cancer nanomedicines, enabling a smoother clinical translation with less failures in the future.

## 1 Introduction

Nanotechnology has generated huge promise in the diagnosis and treatment of cancer. Applications with this technology in oncology range from carriers of chemotherapeutic drugs to radiosensitizers, contrast agents, photothermal therapeutics, and theranostic agents.^1^ Interest in cancer nanomedicines has gained significant momentum in both the public eye as well as the scientific community. Horizon is the biggest supporter of innovative technology in the European Union with a seven‐year budget plan of €80 billion. Nanotechnology is proving to be at the heart of this funding.^2^ In the United States, the National Nanotechnology Initiative has provided at least €1.2 billion annually since 2013.^3^ Despite this funding, there is growing necessity to improve translation as there are many pitfalls in current preclinical assessment.^4^ Immunotoxic effects related to cancer nanomedicines are major clinical roadblocks that must be carefully considered to improve translation in the future.^5^ Nanoparticles aim to reduce immunotoxicities associated with conventional drugs but can also indirectly induce many immunotoxic effects of their own. Their unique physiochemical characteristics (PCC), such as size and large surface area, make them susceptible to undesirable interactions when administered intravenously that can hinder their development as potential cancer nanomedicines. Due to common pitfalls in this area of assessment, and speed at which this technology is growing, immunotoxicity is not fully understood.^6^ There is growing demand to improve current preclinical assessment in order to reduce the amount of failures and increase the number of nanomedicines making it to clinical trials and beyond. It appears there is a need to take a step back from this rapidly growing field to focus on improving the earlier stages of development in order to use this funding to full effect. This will maintain interest in nanomedicines and ensure a long, successful future for cancer nanomedicine research.

This review highlights the broad range of unintentional immunological interactions associated with various nanoparticles researched in the area of cancer nanomedicine. Novel research on nanoparticle immunotoxicity with consideration to regulatory requirements is discussed which is currently advancing knowledge in this area. Improving preclinical testing is essential for the advancement of this technology and must be carefully considered to maximize success.

## 2 Immunotoxicity and Nanoparticles

**Figure 1:**
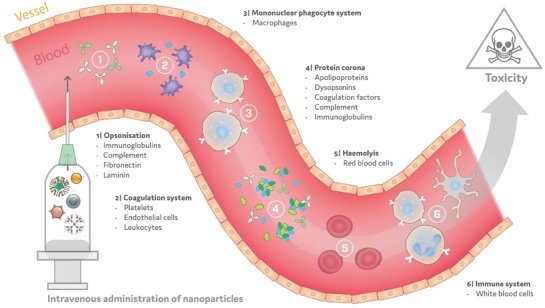
Potential blood component interactions with nanoparticles.

The immunotoxic effects of nanoparticles are broad and vary from acute inflammation^7^ to lung,^8^ liver,^9^ and systemic damage^10^ depending on the nanoparticles composition, structural properties, and administration route.^10^ Upon intravenous (i.v.) administration, nanoparticles will interact with cells and proteins in the blood (**Figure** **1**). Plasma contributes to about 55% of total blood volume (91% water and 9% of proteins and other solutes), erythrocytes are closer to 40%, while white blood cells and platelets making up the rest of the fraction.^11^ Undesirable interactions with each of these blood components pose an immediate threat to the biocompatibility, biodistribution, and efficacy of a cancer nanomedicine.^12^ Moreover, significant alterations to blood components such as those mentioned in Figure 1 may be harmful or beneficial depending on success of design and intended outcome. Nanoparticle PCC play a fundamental role with respect to each of these undesirable interactions and advancing our understanding of how these PCC relate to these effects may be key to ensuring the clinical success of cancer nanomedicines in the future.

Nanoparticles undergo many different interactions when administered i.v. These interactions (some listed within) involve many different cells and proteins found in circulation that can induce a variety of toxic effects on the patient.

### 2.1 Coagulation System

The coagulation system maintains a balance of hemostasis involving clot formation during injury and natural biological inhibitors that inhibit clot formation in healthy conditions.^13^ This delicate balance can be dysregulated by nanoparticles encountering cells and plasma coagulation factors in circulation.^10^ The resulting effects can be severe and even life threatening.^14^ PCC of nanoparticles dictate their interaction with the coagulation system.^15^ If a nanoparticle undesirably interacts with a component of the coagulation system, an activation or inhibition of coagulation factors can disrupt the hemostatic balance either reducing the coagulation response below healthy levels or enhancing coagulation resulting in procoagulant activity.^15^

This disruption is associated with many toxic effects within the coagulation system including disseminated intravascular coagulation (DIC)^16^ which, depending whether it occurs acute or chronically, can induce abnormal hemorrhaging^17^ or intravascular thrombosis^18^ respectively. DIC occurs through depletion of coagulation factors and, if left untreated, can lead to organ failure and even death.^19^ Alterations to the hemostatic balance can be induced directly or indirectly through various mechanisms by nanoparticles including platelet factor upregulation, platelet membrane damage, and endothelial cell interactions (**Table** **1**).

**Table 1:** Nanoparticle subtypes and their various interactions with the coagulation system. Different characteristics of potential cancer nanomedicines have specific effects on the coagulation system. Some examples are listed here. Abbreviations: Generation 7 (G7); disseminated intravascular coagulation (DIC); cadmium telluride (CdTe); iron oxide nanoparticles (IONP); poly(ethylene glycol) (PEG); gold nanoparticles (GNP); mesoporous silica nanoparticles (MSN); trimethylsilane (TMS)

Nanoparticle	Effect on the coagulation system
Dendrimers – Poly(amidoamine), G7, 8.1 nm, cationic)^16^	Rapid fibrinogen binding and aggregation in vitro (100 µg mL^−1^) and complete vascular occlusion phenotype in zebrafish (10 ng).
Dendrimers – Poly(amidoamine), G7, 8.1 nm, cationic)[qv: 19b]	DIC in CD‐1 mice at concentrations more than 10 mg kg^−1^.
Dendrimers – Poly(amidoamine), G7, NH_2_, and FITC functionalized, 8.1 nm, cationic)^20^	Significant increase in platelet aggregation in vitro (whole blood) seen with cationic dendrimers but not neutral (—OH) of anionic (—COOH) (100 µg mL^−1^). Cationic dendrimers could bind directly to platelets and get internalized, leading to changes in cell morphology.
Quantum Dots – CdTe (2.6 and 4.8 nm coated with thioglycolic acid (negative charge) and 2.8 nm coated with cysteamine (positive charge), respectively)^21^	Significant platelet aggregation in vitro through upregulated P‐selectin and GPIIb/IIIa surface receptors along with MMP‐2 stimulated release (3 × 10^−6^ m ).
IONP – Maghemite (22 nm, bare)^22^	Prolonged thrombin time and activated partial thromboplastin time in Sprague Daley rats (0.8 mg kg^−1^).
IONP – Magnetite (4–6 nm, coated in PEG)^23^	Thrombotic occlusion in BALB/c mice at 10 µg kg^−1^ and significant reduction in thrombin time and activated partial thromboplastin time at 0.4 µg mL^−1^.
GNP – (150 nm, SiO_2_‐coated GNP and 2–3 nm bare GNP)^24^	150 nm GNP increased platelet aggregation and 2–3 nm GNP suppressed platelet aggregation in vitro (5 × 10^9^ NP/mL).
GNP – (Colloidal 45 and 85 nm, anionic)^25^	GNP has high affinity for fibrinogen. Significant reduction in clotting time in vitro in both 45 and 85 nm GNP (5 × 10^−9^ m).
GNP – (10 and 50 nm, polyphosphonate coated, anionic)^26^	Reduced clotting time versus PEG and bare GNP in vitro (1.5 × 10^−9^ m concentration for 50 nm GNP and above 70 × 10^−9^ m for 10 nm GNP).
GNP – (20 nm, bare, cationic)^27^	Platelet activation in vitro at 40 × 10^−6^ m with 20 nm GNP. Platelet activation not seen with larger GNP.
MSN – (60–220 nm, 5–15 nm pours)^28^	Pour size, but not nanoparticle size, showed an increasing coagulation potential. A larger pore size bound to more FXII, had a stronger reduction in activated partial thromboplastin time in rabbit blood (2 mg MSN) and a higher haemostatic activity.
MSN – (47.9 ± 7.1 nm, coated with PEG and TMS)^29^	Platelet adhesion and aggregation significantly increased at 100 0 µg mL^−1^.

The coagulation system may act as a hurdle for promising cancer‐associated nanoparticles that could hamper their translation into the clinic due to dose‐limiting effects.^10^ Moreover, circulating tumor cells are known to have a procoagulant phenotype and, therefore, unintentional procoagulant effects may potentially enhance rumor progression by contributing to metastasis.^30^ The range of interactions is broad within this system and varies within specific nanomaterial characteristics for example, polystyrene nanoparticles have been shown to impact on plasma coagulation, but the nature of this interaction depends upon the PCC. Cationic nanoparticles decreased thrombin generation through depletion of factors VII and IX. Whereas anionic nanoparticles activated the intrinsic coagulation pathway.^31^

### 2.2 Opsonization and the Monocyte Phagocytic System (MPS)

When introduced i.v., nanoparticles are immediately covered with proteins from the serum. This surface covering can occur in a dynamic manner referred to as the “Vroman effect,” which explains the competitive absorption of proteins with respect to their concentration, affinity and incubation time.^32^ Once bound, these surface proteins may decide the nanoparticles fate in vivo. This can consequently inhibit a nanoparticle targeting a tumor as surface proteins block the action of targeting moieties on the nanoparticles surface, resulting in increased risk of toxic effects due to off‐site accumulation.^33^ A striking review by Wilhelm et al. in 2016 revealed that after extensively reviewing 10 years of nanoparticle research (2005–2015) only 0.7% of nanoparticles have been successfully delivered to the tumor (median value). Although not adjusting this value for tissue size (i.e., a tumor may be larger than a liver or spleen), this is still a worrying statistic for cancer nanomedicines, and one that puts this technology in a bad light in the public eye. Furthermore, the paper suggested that the MPS and renal system is involved in 99% of this sequestration.^34^ The blood‐containing proteins involved in the MPS are opsonins but can refer to any proteins involved in nanoparticle uptake including non‐specific immunoglobulins and complement proteins.^35^ Opsonins are foremost an immune response, considering nanoparticles as foreign pathogens due to their similarities in size to viruses (which are also in the nanometer range).^36^ When bound they act as signals for phagocytosis and clearance by the MPS.^37^ The complement system plays a critical role in opsonization and uptake of nanoparticles.^38^ This system comprises of more than 30 plasma proteins activated through three different mechanisms: the classical, alternative, and lectin pathways. Each of these mechanisms converges at the point of complement alternative 3 (C3) convertase with cleavage into its active subunits C3a and C3b, the defining step in complement activation.^39^ The alternative pathway can directly induce opsonization of nanoparticles as well as contributing to protein corona formation (discussed next), which activates the classical and lectin pathways for greater complement effect, modifying the nanoparticle surface further.[qv: 35b] Activation of the complement system can result in inflammation,^40^ immune cell activation,^41^ and potentially even tumor growth.^42^

Opsonization can help or hinder a nanoparticle, depending on the intentions of design. The MPS can result in the clearance of nanoparticles reducing their biological effect and increase the dose required for efficacy. It can also be hijacked however, to deliver non‐biodegradable nanoparticles to specific organs (e.g., liver, bone marrow, and spleen)^43^ through the reticuloendothelial system (RES) or sites of inflammation (e.g., cancer, arthritis) which macrophages preferably localize to.^44^ Unfortunately, this phagocytic uptake can also cause healthy tissue damage to the host leading to symptoms like rash, dyspnea, flushing, hypertension, hypotension (pseudoallergic reactions), and even fatal cardiopulmonary syndrome in rare cases.^45^

**Figure 2:**
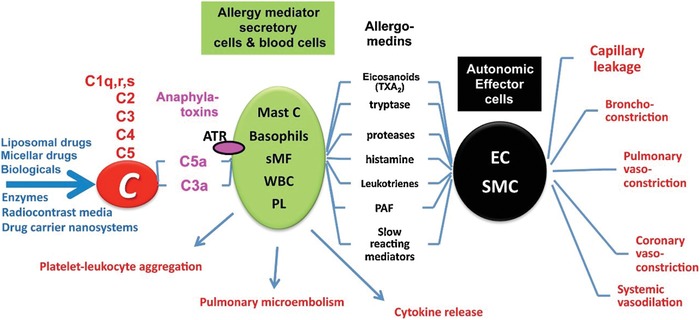
Graphical illustration of the “CARPA cascade” and its associative toxic effects. Abbreviations: C, complement; ATR, anaphylatoxin receptor; Mast C, mast cells; sMF, secretory macrophages; WBC, white blood cells; PL, platelets; EC, endothelial cells and SMC, smooth muscle cells. Reproduced with permission.[qv: 49a] Copyright 2019, Elsevier.

In 2013, the European Medicines Agency (EMA) published reports which commented on safety issues with liposomes,^46^ and iron‐based formulations.^47^ In both cases, infusion reactions were highlighted as issues to be considered in relation to these nanomaterials. In the clinic, liposomes, and iron oxide nanoparticles (IONP) have demonstrated hypersensitive effects which has led to their scrutiny, and has even contributed to the withdrawal of a number of iron oxide nanoparticles from the market.^48^ Complement activation‐related pseudoallergy (CARPA) is an infusion reaction that has been reported extensively for many cancer nanomedicines in addition to liposomes and iron oxide nanoparticles.^49^ The sequence of events that leads to CARPA or the “CARPA cascade” (**Figure** **2**) involves a complex network of cells including white blood cells, platelets, endothelial cells, masts cells, basophils, and macrophages.^50^ Reviewed by Szebeni in 2014,[qv: 49a] CARPA is not sufficiently evaluated at the preclinical stage of nanomedicine development, potentially leading to hypersensitivity reactions occurring in patients during clinical trials resulting in implications for time, money and potentially lives.

#### 2.2.1 Liposomes

Liposomes consist of lipid bilayers with the ability to encapsulate drugs dissolved in solution enabling improved drug stability, reduced toxicity, and improved targeting in vivo. These nanoscale delivery systems have had great success in the area of oncology,^51^ however many studies have reported concerns with liposome–opsonin interactions.

Moghimi and Hunter and later Yan et al. reviewed the role of opsonization with liposomes.[qv: 35a,52] Liposomes are targets for natural antibodies specific for cholesterol and phospholipids found on their surface. This can induce liposome uptake and degradation by macrophages^53^ along with enhancing its association to complement proteins and apolipoproteins. Targeting of liposomes by antibodies can also result in the activation of the complement system. Surface charge, level of saturated/unsaturated phospholipids, and the chemotherapeutic drug (cisplatin/doxorubicin) carried by Doxil (first clinically approved cancer nanomedicine^54^) have each been linked to this activation and found to generate hypersensitive reactions and cardiopulmonary distress in pigs, a model for CARPA in humans.^54^ In addition, the pegylated coating on Doxil is considered a shield against cells and proteins in the serum to prolong the circulation time of nanoparticles. Unfortunately, moderate to severe hypersensitivity reactions still occur in up to 45% of patients.^55^ A later study by Moghimi et al. noted that assumed non‐complement activating liposomes coated with a methoxy poly(ethylene glycol) (mPEG) phospholipid conjugate do, in fact, activate the complement system and thromboxane A2, a thrombotic factor involved in platelet activation and aggregation.^56^ This same paper was able to suppress this activity with the methylation of the phosphate oxygen on mPEG (neutralizing the negative surface charge).^57^ Recent papers have pinpointed these PEG‐associated effects to naturally occurring anti‐PEG antibodies detected in 44.3% of healthy human donors.^58^ This matches up well with the values suggested for hypersensitivity reactions mentioned previously. Beyond hypersensitive effects, these antibodies have also been associated with accelerated drug clearance with repeated doses of PEG‐coated drugs resulting in reduced efficacy in the long term.^59^ A companion diagnostic test may be useful here to detect good responders to this treatment; However, as a recent paper points out, the levels of anti‐PEG antibodies alone may not be efficient predictors, but their affinity, epitope specificity, and antibody class may also be necessary inclusions for such a test, in addition to establishing the serum levels of complement inhibitory molecules in the individual.^60^ As referred to previously, Szebeni[qv: 49a] has highlighted concerns for a host of liposomal formulations and CARPA, including Doxil, Myocet, DaunoXome, and Visudyne, which have all been used in the treatment of various cancers.^54^, ^61^

#### 2.2.2 Gold

Gold nanoparticles (GNP) are proving to have a variety of applications in cancer treatment and diagnosis. These include imaging,^62^ photothermal agents,^63^ nanocarriers,^64^ and radiosensitizers.^65^ Clinically, silica‐cored gold nanoshells have shown optimism in treating head and neck and prostate cancers through hyperthermia,[qv: 61c] and gold nanocarriers of nucleic acids and TNF‐α are currently in trials for gliobastoma^62^ and various solid tumors,^63^ respectively.

Dobrovolskaia et al. evaluated the proteins absorbed to the surface of citrate‐stabilized GNP (30 and 50 nm).^66^ Fibrinogen was discovered to be the most abundant protein on GNP. This led to coagulation assessments which identified no change in coagulation time or platelet aggregation. It is not yet known if nanoparticles are capable of adsorbing significant levels of fibrinogen (or any serum protein for that matter) to toxic concentrations (<1.0 g L^−1^ in the case of fibrinogen^67^), before an alternative dose‐limiting effect occurs. Moreover, C3 was also found on GNP but showed no activation of the complement cascade through either the classical or alternative pathways. Recently, Quach and Kah evaluated the complement activation of pegylated gold nanospheres, nanostars, and nanorods.^68^ With the use of human whole blood, along with C1q‐ and C4‐depleted serum, it was discovered that each gold nanomaterial could activate the complement system through all three complement pathways. Subsequent phagocytosis through U937 promonocytic cells was also observed, which could lead to their clearance in vivo. Pegylation in this case reduced complement adsorption but was unable to prevent complement activation. This same group came to a similar conclusion a year later where they noted that spherical, pegylated GNP had reduced—but not prevented‐ complement activation over citrate‐capped GNP. This decreased complement activation was found to be highly correlated to the lower levels of pegylated GNP internalized into human promonocytic cells over citrate‐capped GNP.^69^ Moreover, this group also looked at the effect of various shapes (spherical and rod‐shaped), coatings (different charges and hydrophilicities) and sizes (spherical diameter of 20 and 40 nm; cylindrical diameter of 40 nm and length of 10 nm) of GNP on complement activation. The larger the surface area and greater the positive charge was found to be the biggest inducers of complement activation in this case.^70^

#### 2.2.3 Iron oxide

IONP uptake by the MPS has been utilized in the clinic as a method of imaging cancer through the so called “Trojan‐horse” delivery. Lymph node metastasis along with bowel and liver cancers have been successfully viewed in the clinic using this method.^71^ In the last 10 years, however, four initially successful IONP have been discontinued in the clinic by either the FDA (U.S. Food and Drug Administration) or EMA: Combidex in 2007, Feridex in 2008, Resovist in 2009, and Gastromark in 2012.^48^, ^71^ CARPA and severe anaphylactic reactions has been a noted side effect with clinically approved IONP and are one of the major reasons behind these withdrawals.^48^ An EMA report in 2013 and a subsequent FDA report two years later discussed the recommendations to manage allergic reactions with intravenous iron.[qv: 47,49b,72] To limit potentially fatal hypersensitivity reactions upon treatment with intravenous iron, particular attention should be given during and at least 30 min after infusion, with appropriate treatment administered if reactions occur. Superparamagnetic iron oxide (SPIO) nanoworms were tested for their complement‐dependant uptake by monocytes, neutrophils, lymphocytes, and platelets in normal and tumor‐bearing mice and humans.[qv: 37b] Nanoworm uptake was measured through magnetically labeled cells containing the SPIO with and without the action of the complement system (ethylenediaminetetraacetic acid (EDTA) and antiproperdin were added to the blood as complement inhibitors). Results showed C3‐dependant recognition of the nanoworms by leukocytes and platelets in both normal and tumor bearing humans and mouse blood. Feridex showed complement‐related toxicities in clinical use and in vivo studies confirmed complement‐related leukocyte uptake.^73^ Literature related to this reaction and IONP is well established. A large amount of papers report complement activation with dextran coatings, which is common on clinically approved IONP.[qv: 38b,74] Wang et al. discovered that dextran SPIO nanoparticle activated complement primarily through the alternative pathway (AP) in human serum.^75^ Alcohol groups on the sugar were modified with alkyl, acyl, and cross‐linkers in an attempt to reduce/prevent AP activation. Complement activation and C3 opsonization was still observed and therefore activation was independent of the alcohol groups on dextran. Iron–dextran ratios were also studied with little difference in the activation observed from this perspective either. The AP pathway could be exploited in order to alleviate anaphylactic affects with IONP in the clinic.

#### 2.2.4 PAMAM Dendrimers

Generation 5 (G5) dendrimers with amine and hydroxyl groups along with G4.5 dendrimers that are carboxyl functionalized have shown to inhibit complement activation through regulation of γ‐globulin, affecting its secondary structure.^76^ There is very little literature that details any further interactions with this system, and so more work is required in this space.

CARPA is one of the most significant immunotoxicity challenges for cancer nanomedicines in the pipeline, contributing to the clinical withdrawal of a host of IONP and resulting in the release of safety reports for both liposomal and iron oxide nanoformulations by EMA. Recent papers have been published, however, which go into great depth on the influence of different nanoparticle shapes, surface areas, coatings and charges on complement activation, and offer suggestions to alleviating this activity.^70^, ^77^ Furthermore, although the molecular events leading to CARPA are yet to be fully established, the mechanisms involved are becoming a lot clearer, with the importance of the formed protein corona on nanomaterials (Section 2.3) and its interaction with immunoglobulins now understood as a significant event leading to complement activation.^78^ In the future, there will likely be a companion diagnostic test to detect good responders to various nanomedicines. Additionally, the advancements made in the early detection of CARPA with nanomedicines will certainly reduce this effect occurring in the clinic in the future.

### 2.3 Protein Corona

The protein corona is an expansion on opsonization and refers to the biological identity of nanoparticles and all the possible blood proteins bound to their surface. These proteins include apolipoproteins, dysopsonins, and coagulation factors as well as complement and immunoglobulins.^79^ Stimulation and suppression of the immune system may be an indirect effect of protein corona formation leading to immunotoxic effects.^80^ Literature has shown the corona to vary between cancers^81^ and patients,^82^ which makes it difficult to implement a design strategy for avoiding/inducing specific coronal interactions. Opinions on the overall influence and manipulation of the protein corona are varied with arguments for and against existing in the literature.^83^ More high‐power studies are required to find the true extent of this alteration and whether this is significant enough to vary nanoparticles in vivo safety and efficacy between patients.^84^ If this is the case, patient‐specific design could become a future consideration. A notable study by Caputo et al. evaluated the corona formed on lipid nanoparticles in 25 human serum samples (20 with pancreatic cancer and 5 without) and, using SDS‐PAGE evaluation, gave correct discrimination of the cancer in 88% of cases.^85^ Therefore, in vitro assessments with patient‐derived blood samples may have diagnostic value as well as a potential predictor for treatment outcome, which could allow for treatment interventions on expected immunotoxicities derived from these nanomedicines, improving their safety and efficacy.

A small summary of the immunotoxic effects derived from corona proteins is given by Lee et al.^79^ Surface chemistry seems to be the underlying PCC involved in the variety of protein coronas established. It is important to note that both opsonization and the protein corona have the potential to improve the efficacy of a cancer nanomedicine with regards to tumor targeting^86^ and avoiding immunotoxic effects.^87^ This suggests the full potential of a nanomedicine may not be established at all, or until late preclinical/clinical evaluation if not sufficiently assessed. Therefore, despite arguments for and against the protein corona in the literature, gaining early knowledge of this in vivo modification can only benefit a nanomedicines development. Standardized assays for the preclinical evaluation of the protein corona in relation to the safety and efficacy of cancer nanomedicines seem to be a long way away at present. Benefits to this avenue could prove significant for future translation.

### 2.4 Hemolysis

Hemolysis refers to the damage of red blood cells (RBC), which induces the release of intracellular proteins like hemoglobin into the blood.^88^ Damage and protein release by RBC is associated with anemia and renal failure.^89^ Research on the mechanisms behind hemolysis with nanoparticles is scarce in the literature although a necessary hurdle to overcome during toxicity testing.^90^

#### 2.4.1 Dendrimers

Dendrimers are being researched in many aspects of oncology including drug, gene, and antibody delivery,^91^ diagnostics through magnetic resonance imaging (MRI) contrast agent delivery[qv: 91b] and photodynamic therapy.^92^ In an early study, cationic dendrimers were shown to be bigger inducers of hemolysis than their anionic counterparts.^93^ The negatively charged surface of RBC means they have high affinity for cationic NPs.^94^ In 2010, the hemolytic role of polyamidoamine (PAMAN) dendrimers was evaluated and deemed to occur in a dose and time‐dependant manner due to an increased release of hemoglobin from RBC. Interestingly, this study also concluded that human serum albumin had a protective role for hemolysis, suggesting that when bound to the surface of the dendrimer, membrane damage is decreased.^95^ Similarly, Wang et al. evaluated PAMAN dendrimer interaction with human RBC and found similar results.^96^ Concentration dependant RBC membrane damage, morphology alterations and hemolysis was observed with PAMAN dendrimers (0.1–5 mg). This effect was reduced and even diminished with the addition of PEG chains (5K and 20K) to the dendrimer surface. This is currently a major hurdle for the clinical translation of cationic dendrimers, however, advancements in modifying their positively charged surface to reduce or eliminate this interaction is increasing their promise.^97^

#### 2.4.2 Iron Oxide

Maghemite nanoparticles were evaluated for their potential hemolytic properties in vivo.^98^ These IONP were introduced at three different doses (7.5, 15, and 30 mg per kg per body weight) weekly for 28 days into Wistar rats. Blood samples were taken weekly and hematological analysis was undertaken for each sample for the given time period and dose. Results indicated IONP induces oxidative stress leading to an inflammatory response, which causes cytotoxic stress to RBC as well as modulating the immune system by increasing the levels of white blood cells (monocytes and neutrophils) in circulation. Furthermore, reductions in antioxidant enzymes (superoxide dismutase, catalase, and glutathione) in a dose and time‐dependant manner suggest reactive oxygen species (ROS)‐mediated oxidative damage is the major mechanism behind observed cytotoxic effects. In the same year, Ran et al. comparably showed that bare magnetite (60–90 nm) could induce eryptosis both in vitro (25 µg mL^−1^) and in vivo (25 mg kg^−1^).^99^ Dose and time‐dependant increases in ROS was equally observed in this study.

#### 2.4.3 Gold

Aseichev et al. investigated erythrocyte hemolytic effects of 5, 10, 20, 30, and 60 nm GNP.^100^ Prior to this, very few studies had been done evaluating gold nanoparticles hemolytic potential. Using the levels of hemoglobin in the blood and erythrocyte osmotic resistance as markers, hemolysis was evaluated on uncoated and plasma‐component coated GNP. Results showed that selected concentrations of GNP (≈<20 × 10^−6^ m of gold) coated or uncoated, showed no erythrocyte hemolytic activity; However, smaller GNP (5, 10, and 20 nm) showed increased hemolytic effects at 20 × 10^−6^ m than their larger counterparts, highlighting GNP size and dose are major parameters involved in interactions with RBC. A previous study by Love et al. concluded positive and negative‐charge 30 nm GNP induced hemolysis in a concentration‐dependant manner (both significant at 50 µg mL^−1^). Additionally, this was found to occur in a ROS generation‐independent manner, suggesting other cellular destruction mechanisms must be at play.^101^

#### 2.4.4 Quantum Dots

Quantum dots (QD) have shown great promise preclinically in the diagnosis of cancer through the imaging of tumor antigens, sentinel lymph nodes, and even circulating tumor cells. Questions remain, however, on the safety of these flexible probes, as they have yet to progress into clinical evaluation.^102^ A study examining the hemolytic activity of QD came to the conclusion that nanoparticle size induces varying hemolytic effects. Three mercaptosuccinic acid‐capped QD differing mostly on size were found to have differing hemolytic mechanisms and adversities (**Table** **2**). Green‐emitting QD causes hemagglutination, while yellow‐emitting QD caused slight hemolysis. Both of these QD were found to have strong affinity for cellular glycocalyx, a glycoprotein‐polysaccharide located on RBC plasma membrane.^103^ By contrast, the red‐emitting QD induced a heavier hemolytic response and was found to modify the lipid conformation on RBC through phosphate ester bond breakage.^104^

**Table 2:** Three quantum dots experimented for their role in hemolysis. Quantum dots used in the study by Wang and Jiang^104^

Quantum dot	λ_abs_ ^a)^ [nm]	λ_em_ ^b)^ [nm]	*Z*‐potential [mV]	Diameter [nm]
Red	595	624	−18.58 ± 1.02	5.4 ± 0.2
Yellow	544	568	−22.48 ± 0.78	3.5 ± 0.3
Green	530	549	−22.72 ± 2.23	2.3 ± 0.2

^a)^ Typical absorption band

^b)^ Emission maximum.

#### 2.4.5 Mesoporous Silica

Mesoporous silica nanoparticles (MSN) have been researched as promising multifunctional tools in cancer acting as drug carriers for improved targeted therapy and imaging.^105^ Hemolysis, however, has resulted in the rapid decline of MSN application in the clinic due to silanol groups on the outer surface of the nanoparticle inducing an adverse hemolytic effect.^106^ The silanol surface of the silica nanoparticles (≈600 nm) has been shown to associate to phosphatidylcholine molecules present on the surface of RBC inducing structural alterations to the cell membrane resulting in internalization of the nanoparticle and lysis of the cell. This method of hemolysis is avoided, however, with MSN closer to 100–200 nm. Furthermore, masking the silanol groups reduces silica nanoparticle hemolytic activity.^107^ Interestingly, further studies concluded that the protein corona had a protective role for MSN‐related hemolysis.^108^ Plasma‐containing proteins hemoglobin, serum albumin, and hemolysate were found to have a hemolytic suppressive role for MSN. The mechanisms behind this suppression are yet to be identified.^106^

### 2.5 Immune System

Nanoparticles are engineered to be either immunostimulatory (e.g., nanovaccines), immunosuppressive (e.g., anti‐inflammatory) or aim to avoid the immune system altogether (e.g., pegylated nanoparticles). Unintentional immunomodulation can occur which results in immunotoxicity for the patient and reduces the efficacy of the nanomedicine. Endotoxin contamination is a major factor to consider with immune modulation of any nanomedicine and is discussed further in Section 3.2.

#### 2.5.1 Immune Stimulation

Undesirable immune stimulation involves the unintended activation of the immune system by nanoparticles (**Table** **3**). Side‐effects related to this include hypersensitivity, inflammation, and anaphylaxis.^7^ Cytokines are proteins released by cells which can regulate immune responses like inflammation.^109^ Excessive inflammation can be damaging to the body and hence cytokines are biomarkers for immunotoxicity with nanomedicines.^110^ The toxicities associated with modified cytokine production can range from mild (fever and nausea)^110^ to fatal organ damage.^111^ Cytokine production induced by nanoparticles can result in the recruitment of proinflammatory cells such as macrophages, dendritic cells, and neutrophils.^112^ Nanoparticle localization in the lung can induce the recruitment of these inflammatory cells, which aids in the upregulation of ROS and cytokines potentially damaging the lung tissue and causing lung diseases like asthma and bronchitis.^113^ Nanoparticle‐induced inflammation can also lead to liver and kidney tissue damage.^114^ Moreover, inflammation in now a well‐established hallmark of cancer but it is not know yet if anticancer drugs that indirectly induce proinflammatory effects have their efficacy reduced in vivo or even display tumor progressive effects.^115^

**Table 3:** Immunostimulatory effects of nanoparticles associated with oncological research. Nanoparticle characteristics are described in the left column while their associated immunostimulatory effect is summarized on the right

Nanoparticle	Immunostimulatory effect
GNP (2 nm core, neutrally charged and coated with tetra(ethyl glycol), hydrophobic zwitterion or hydrophilic zwitterion)^116^	Hydrophobic zwitterion coating produced an anti‐inflammatory responses in vitro (100 × 10^−9^ m) and proinflammatory response in vivo (2.75 mg NP/kg). Tetra (ethyl glycol) coating produced an anti‐inflammatory response‐ all measured based on TNF‐α levels. Hydrophilic zwitterion coating showed minimal changes.
GNP (10 and 50 nm, uncoated)^117^	After one day, increase in IL‐1β mRNA and IL‐6 in the liver after 10 and 50 nm injected into rats (22 µg Au/kg). Increase in IL‐6 and TNF‐α with 50 nm GNP in kidney after one day. After five days, these proinflammatory effects normalized.
IONP (Resovist, carboxy dextran coated, 5 nm)^118^	Resovist (20 µg Fe) induced a phenotypic shift in THP1 derived M2 macrophages to M1‐like macrophages in vitro.
IONP (magnetite, coated with polyacrylic acid, 10.1 ± 2.4 nm)^119^	Significant increases in neutrophils and small and large lymphocytes after injection into PD‐1 mice (50 mg kg^−1^ IONP).
IONP (coated in phospholipid, 37–43 nm hydrodynamic size)^120^	White blood cells were significantly increased in ICR mice (2–4 mg kg^−1^ IONP). In particular, neutrophils and eosinophils were increased. Lactate DeHydrogenase, IL‐6, and IL‐8 levels were elevated. Suppression of antigen presentation proteins and dendritic cell maturation were also observed.
IONP (maghemite, needle‐like shape, (diam = 50–200 nm; length = 10 nm)^121^	ICR mice were treated (by intratracheal injection) with 0.5‐2 mg kg^−1^ IONP/ body weight. Neutrophils and lymphocytes significantly increased in the lung at 2 mg kg^−1^. Th1 polarization was induced in the lung.
Liposome (cationic, 107 and 223 nm), coated with 1,2‐dioleoyl‐3‐trimethylammoniumpropane, cholesterol and phosphatidylcholine)^122^	Cationic liposomes induced a significant increase in Th1 expression of IL‐2, IFN‐γ, and TNF‐α (C57BL/6 mice at 20 mg kg^−1^ NP). These liposomes were also shown to activate the immune system in a TLR4‐dependant manner.
Dendrimer (polyamidoamine, generations 0–3)^123^	CD‐1 mice were treated with 100 and 500 µg mL^−1^ into murine air pouches. A dose‐dependent rapid influx of neutrophils, in particular, was observed. IL‐10 and IL‐1Ra expression is also increased.
Dendrimer (polyamidoamine, generations 4 (6.2 ± 0.3 nm), 5 (7.5 ± 0.3 nm), and 6 (10.3 ± 0.4 nm))^124^	Dose‐dependent and amino group number‐dependent increase in MIP‐2, TNF‐α, and IL‐6 in macrophages (0.2–1.2 µm).

#### 2.5.2 Immune Suppression

Unintentional immune suppression by nanomedicines can contribute to the development of immunotoxicities such as an anti‐inflammatory environment, myelosuppression, and reduced immune response to cancer.^125^ Most immunosuppression evaluations with nanomedicines occur indirectly and are established in vitro. As before, cytokine and chemokine levels are the main method used in the literature to test immune suppression preclinically (**Table** **4**).

**Table 4:** Immunosuppressive effects of nanoparticles associated with oncological research. Nanoparticle characteristics are described in the left column while their associated immunosuppressive effect is summarized on the right

Nanoparticle	Immunosuppressive effect
GNP (7.4 nm ± 2.8 nm, uncoated)^126^	Significant dose‐dependent reductions in leukocyte migration to the peritoneal cavity and significant reductions of IL‐1β and TNF‐α in peritoneal fluid of mice (700, 1000, and 150 µg NP/kg).
GNP (21 nm, uncoated)^127^	Significant reduction of TNF‐α and IL‐6 mRNA expression in adipose tissue macrophages in mice (7.85 µg NP/g).
GNP (10–15 nm, uncoated)^128^	Significant reduction in endotoxin induced nitric oxide upregulation in macrophages (in vitro) in a dose dependant manner (up to 40 ng mL^−1^ GNP).
GNP (size ranged from 4 to 45 nm, pegylated and fluorescein‐tagged)^129^	4 nm GNP is most potent inhibitor of TLR9 in macrophages in vitro (up to 40 µg mL^−1^ GNP).
IONP (10 and 30 nm, both coated with oleic acid and amphilic polymer)^130^	Indirect anti‐inflammatory effect with monocytes. Endotoxin adsorbed to IONP surface inhibiting TLR4 and CD14 signaling. NF_K_B signaling is also deregulated (1–100 µg mL^−1^).
IONP (200 and 240 nm hydrodynamic diameter, coated with starch and PLGA, respectively)^131^	IL‐6 secretion is significantly reduced in primary monocytes by both IONP (500 ng mL^−1^).
IONP (Resovist, 58.7 nm hydrodynamic diameter, coated with carboxydextran)^132^	Delayed‐type hypersensitivity (DTH) was reduced by Resovist (0.2–10 mg Fe/kg) with ovalbumin‐challenged BALB/c (a model for DTH). A significant reduction in IFN‐ϒ and increase in IL‐4 suggested a shift from Th1 to Th2. A reduction of macrophages, IL‐6, and TNF‐α was also observed at the injection site.
IONP (coated with poly(vinylalcohol) and fluorophore, 29.4 ± 4.1 –to 122.1 ± 14.6 nm)^133^	Decreases in monocyte‐derived dendritic cells ability to process antigen and activate CD4^+^ T cells (20 µg mL^−1^ IONP).
Liposome (Doxil)^134^	BALB/c mice with C26 subcutaneous tumor were injected with 2.5–20 mg kg^−1^ of Doxil or doxorubicin. At high doses, clearance saturation is achieved due to suppression of the MPS, prolonging Doxil circulation time.
Quantum dot (CdSe/ZnS, carboxyl terminated, 8 nm hydrodynamic diameter and 655 nm max emission)^135^	Decreased phagocytic function and viability of macrophages in vitro (2.5 × 10^−9^ m). Decreased viability to lymphocytes in vitro (2.5 × 10^−9^ m). Damage to spleen lymphocytes in BALB/c mice (2 × 10^−9^ m kg^−1^).
Dendrimer (polyamidoamine, generation 3.5, glucosamine‐conjugated)^136^	Deceased levels of IL‐6, IL‐1β, TNF‐ α, IL‐12, MIP‐1a, and MIP‐1b in macrophages and dendritic cells (200 µg mL^−1^).

Thorough in vitro and in vivo assessment is required for sufficient testing of immunomodulating characteristics of nanoparticles. Immunotoxicity of a nanomedicine may only be fully established in vivo so particular attention to animal model selection is important. This should be decided depending on the question you want to answer based on preliminary in vitro data and information on similar nanomedicines described in the literature. Nanoparticles can display multiple immunoregulative roles in vivo. For example, Toll‐like receptor (TLR) 9 was found to be downregulated while TLR4 upregulated in two different studies with silica oxide nanoparticles.^137^ Preclinical studies must be designed with caution as immunological responses can vary between in vitro and in vivo systems, and the gaps between these systems can be large[qv: 6a] (**Table** **5**). Moreover, immunotoxic studies without consideration to endotoxin contamination within the nanoformulation offer little to the reader in attempting to understand these associated effects.

**Table 5:** Summary of the potential immunotoxicities associated with promising cancer nanomedicines. Common cancer nanomedicines and their most researched oncological applications are described above, along with their associated immunotoxicities based on preclinical and clinical evidence (if applicable). Importantly, much of the literature in this space can be contradictory due to the variety of PCC tested for each nanoparticle along with the large array of assays currently used in this space. Moreover, some of these nanomaterials have been largely understudied for particular immunotoxicities. It is therefore important to only use this evidence as a guideline of what to expect, putting the most weight on similar nanoparticles that have been clinically tested

Nanomaterial	Oncological applications	Potential immunotoxicities
Liposomes	Drug delivery	Clinic: Induction of hypersensitive reactions by pegylated liposomes. In vivo: Immune stimulation with Doxil. In vivo: Immune suppression with cationic liposome.
IONP	Imaging Magnetic hyperthermia	Clinic: Induction of hypersensitive reactions by IONP of various coatings. In vivo: Prolonged and reduced thrombin time and activated partial thromboplastin time by IONP of different surface chemistries. In vivo: Hemolytic potential with maghemite and magnetite IONP. In vitro and in vivo: Immune stimulation and immune suppression from IONP of various PCC.
GNP	Photodynamic therapy Radiosensitizer Drug delivery Imaging	In vitro: Reduction in clotting time with anionic GNP. In vitro: Size dependant platelet aggregation and activation. Ex vivo: Complement activation associated with a positive charge and large surface area. In vitro: Dose‐dependent hemolytic potential. In vitro and in vivo: Immune stimulation and immune suppression with GNP of various PCC.
Dendrimers	Drug delivery Imaging Photodynamic therapy	In vitro and in vivo: Strong coagulation potential with cationic dendrimers In vitro: Hemolytic responses with cationic dendrimers. In vitro and in vivo: Polyamidoamine dendrimers of different generations and surface groups display an immune stimulatory response in vivo (amino surface) and an immune suppressive response in vitro (glucosamine surface).
MSN	Drug delivery Imaging	In vitro and in vivo: Coagulation potential. In vitro: The number of silanol groups on the surface of MSN is related to the level of hemolysis induced.
QD	Imaging	In vitro: Platelet aggregation by positive and negatively charged cadmium‐telluride QD. In vitro: Hemolysis properties of negatively charged mercaptosuccinic acid‐capped QD in a size‐dependant manner.

### 2.6 Role of the Active Pharmaceutical Ingredient in Immunotoxic Effects

Although not discussed at length here, the active pharmaceutical ingredient (API) of a nanomedicine will most likely still display its inherent immunotoxic effects despite being incorporated into a nanoformulation aiming to reduce or eliminate these.[qv: 6b,138] In these cases, immunotoxic effects of both the nanomaterial and API should be considered individually and collectively when weighing potential risks. This is necessary as the API may accumulate in sites foreign to what is observed in the clinic (i.e., lymphatic system or spleen), resulting in unforeseen off‐target side‐effects. Additionally, one component of the nanoformulation may exacerbate the toxic effects of another. For example, Abraxane (albumin bound paclitaxel) has higher incidences of neuropathy than paclitaxel alone,^139^ and Caelyx (liposomal doxorubicin) induces hand and foot syndrome at a higher rate than doxorubicin alone.^140^ The differences in toxic effects between clinically approved nanomedicines and their API versus their API alone is described in Brand et al.^141^

## 3 Improving Immunotoxicity Assessment

### 3.1 Endotoxin Contamination

Endotoxin is a component of gram‐negative bacteria cell walls and a potent nanoparticle contaminant. Endotoxin poses a real treat to cancer nanomedicine development as low levels of the contaminant is known to drive immune stimulation, promote inflammation, tissue damage, and even septic shock.^142^ Picogram levels of endotoxin have been shown to induce proinflammatory cytokine expression in vitro,^143^ while human trials suggest a procoagulant^144^ and immunoregulatory^145^ profile. Endotoxin has also been shown to play a role in activating the complement system^146^ and inducing hemolysis.^147^ Therefore, it may play a role in the immunotoxicities described here.

Nanomaterials are thought to be particularly vulnerable to endotoxin binding due to their large surface‐to‐volume ratios. The available lipid moiety and phosphate group on endotoxin allow it to bind to both hydrophobic and positively charged surfaces on nanoparticles.^148^ Furthermore, this contaminant is highly stable against conventional sterilization techniques such as autoclaving. Dobrovolskaia and McNeil reported in 2013 that more than 30% of nanomaterials fail early in preclinical studies due to endotoxin contamination based on nanomaterials evaluated by the US Nanoparticle Characterization Laboratory (US‐NCL).^149^ Moreover, endotoxin contamination has been shown to vary between batches of the same nanomaterial produced from the same lab.^150^ A lack of consistency and an absence of good manufacturing practice with nanomedicine synthesis could be detrimental for their translation.

Considering the regulatory position on this matter, cancer nanomedicines have been approved as both drug products and medical devices. Both of these categories have their own established regulatory paths and endotoxin limits agreed between the FDA and EMA. To control for the varied potency between different endotoxins, a universal unit of measurement is usually accepted—EU or endotoxin units. In the research setting, 100 pg of endotoxin can be approximated to 1 EU.^151^ The endotoxin limit for individual drug products that make contact with the circulatory system is described by the formula K/M. Where K is 5.0 EU per kg of body weight and M is the maximum recommended dose of product per kilogram of body weight introduced in a single 1 h period (5 EU kg^−1^ h^−1^). By contrast, a medical device has an endotoxin limit of 20 EU at 0.5 EU mL^−1^.^151^, ^152^

**Figure 3:**
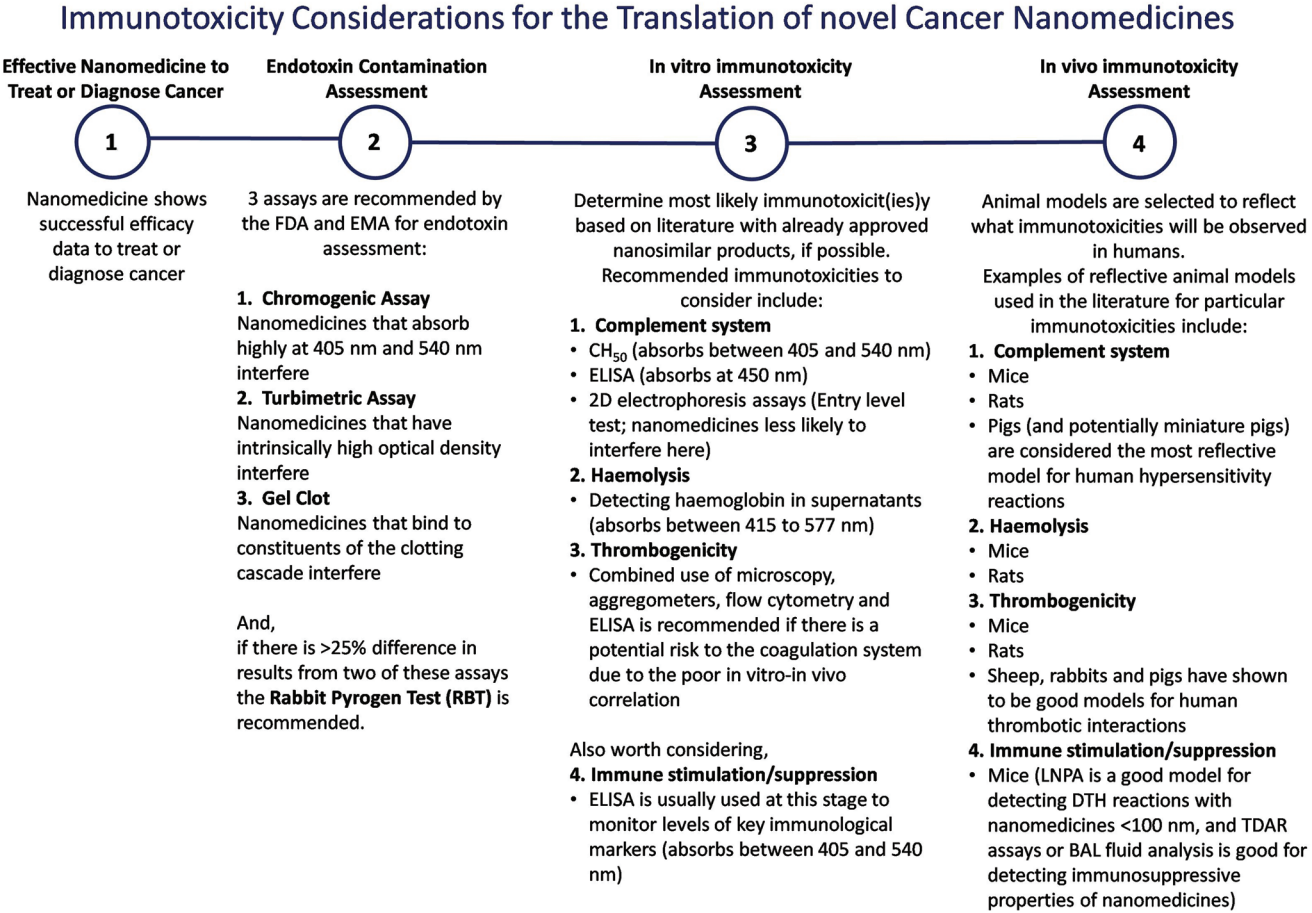
Summary of considerations for endotoxin, in vitro immunotoxicity and in vivo immunotoxicity assessment.

Conventionally, accepted endotoxin contamination evaluation assays (such as Limulus amoebocyte lysate ‐LAL assay) have been challenged in the literature. One LAL assay has been suggested as inadequate to quantify endotoxin levels with two LAL formats being advised.^5^, ^151^ If there is a 25% discrepancy in levels between assays, the rabbit pyrogen test is recommended (**Figure** **3**).^5^ These assays can inform the researcher whether to chase further endotoxin purification, re‐attempt to optimize the synthesis procedure, alter nanoparticle design or bring the nanomedicine forward to in vitro and in vivo studies. One major issue to consider is nanoparticle interference with these assays is common and related to their absorbance wavelength and interaction with constituents of the assays.^90^ It is suggested that choice of LAL assay should be considered in a case‐by‐case basis for each nanomaterial.^151^ A recent review by Li and Boraschi^153^ discussed the different methods of detection and elimination of endotoxin in nanoformulations. From this review, it is emphasized that our best chance for limiting endotoxin levels in nanoformulations comes from careful consideration for endotoxin contamination during the synthesis procedure rather than eliminating the contaminant later on in the testing process. Readjusting synthesis techniques and using endotoxin‐free reagents has been shown to significantly reduce endotoxin in nanoformulations.^154^

As discussed here and in a recent paper by Li et al.,^155^ there is a serious lack of consideration to potential endotoxin contamination in the literature when it comes to the assessment of immunotoxicity with nanoparticles. Very little of the available literature includes an endotoxin contamination assessment, making interpreting these results extremely difficult. Remarkably, of the 63 papers depicting immunological effects with nanomedicines described previously, only 8 papers (12.7%) included any endotoxin contamination assessment of their nanomedicine (values exclude papers testing already clinically approved cancer nanomedicines). Furthermore, endotoxins wide range of signaling means it can give false positive/negative results on a wide array of immunological pathways. It is therefore necessary to assess endotoxin contamination before any immunological assessment to avoid these common mistakes.

### 3.2 Sterilization and Depyrogenation

Further considerations to avoiding contamination in nanoformulations involve sterilization and depyrogenation. The current recommended sterility assurance level of a final product is 10^−6^,^156^ meaning for a nanoformulation to be sterile there can be no more than one viable microorganism in one million parts of the final product. Many methods of sterilization and depyrogenation are available but can potentially alter the PCC of nanoparticles. Vetten et al. reviews these depyrogenation and sterilization methods and concludes that there is not one single method sufficiently capable of purifying all nanoparticles and each nanoformulation must be considered in a case‐by‐case basis.^157^

### 3.3 In Vitro Immunotoxicity Assays

As discussed above, there is a broad spectrum of interactions with nanoparticles when introduced into the blood circulation. It is necessary to review the literature (paying particular attention to approved nanomedicines) and have a calculated prediction on where the particular nanomedicine in question holds the highest immunotoxic risk, and decide on in vitro and in vivo models from there. For example, CARPA may be the biggest threat to liposomes, while hemolysis is a major risk factor for cationic dendrimers. Moreover, nanoparticles interfere with conventional assays for drug immunotoxicity so it is also advised to select assays in which nanomedicines are less likely to interfere (i.e., low absorbance at wavelength in which the assay is measured) or aim to get over the assay interference (i.e., magnetically separating iron oxide nanoparticles before measuring end‐point, centrifugal separation of nanoparticles from end solution, or modifying the wavelength measured within the assay). Particular selection to the assays that have the strongest clinical relevance for the given nanomedicine is essential (Figure 3). The European Nanomedicine Characterisation Laboratory (EUNCL) and the US‐NCL are harmonizing nanoparticle in vitro assessment for a more standardized immunotoxicity analysis (see Section 4).[qv: 6a,158] CARPA, hemolysis, and thrombotic potential are considered the major immunotoxicities to evaluate at in vitro stage.^5^

#### 3.3.1 Complement Activation

To assess complement activation with nanoparticles in vitro, three common methods are used in the literature—CH_50_ hemolytic assay,^159^ enzyme‐linked immunosorbent assay (ELISA),^75^ and 2D gel electrophoresis.^160^ CH_50_ assay is quick but hemolytic nanoparticles and nanoparticles with high absorbance between 405 to 540 nm can interfere with the assay. ELISA can screen many nanoparticles in one test but nanoparticles with high absorbance at 450 nm can interfere. Nanoparticles may be less likely to interfere with gel electrophoresis, but this technique is semiquantitative. Therefore, it is suggested that gel electrophoresis be used as a preliminary test for complement activation; a positive result from this test will lead to further tests to measure the full extent of this activation.

#### 3.3.2 Hemolysis

The method most utilized in the literature to assess hemolysis is detecting hemoglobin in supernatants after treatment with nano‐particles using a spectrophotometer.^161^ This method is most effective for nanoparticles that have low absorbance at 540 nm (if detecting cyanmehemoglobin). Alternatively, measuring oxyhemoglobin is also is also a possibility. In this case, interference is observed mostly with nanoparticles with high absorbances between 415 and 577 nm.^162^ Oxidative and mechanical stress to red blood cells are also markers for hemolysis that have been evaluated in the literature to a lesser extent.^163^

#### 3.3.3 Thrombogenicity

Platelet activation/aggregation, coagulation time, and factor release are the major properties assessed when identifying nanoparticles interaction with the coagulation system. For these experiments, most techniques involve combinational use of microscopy, aggregometers, flow cytometry or ELISA.^20^, ^21^, ^26^ Different techniques will answer different questions, so it is important to decide whether a quick answer on clotting time is sufficient, or you wish to decipher the molecular mechanisms behind an observed response.

#### 3.3.4 Immune Stimulation/Suppression

Beyond these immunotoxicities, immune stimulation/suppression and the MPS are further considerations, in particular for cancer nanomedicines (unintended myelosuppressive properties are observed to a far lesser extent with the cancer nanomedicines described here, and so would not be described in detail. For more information on evaluating myelosuppression, see ref. 164). Proinflammatory cytokines and leukocyte proliferation are widely used in the literature as markers for immune stimulation and suppression.^110^, ^165^ The interaction between nanoparticles and the MPS is quite well established in the literature and advancements in immune‐evading coatings and nanoparticle PCC enable predictions of in vivo biodistribution quite effective.^166^ In vitro models for evaluating MPS are always advancing.^167^

#### 3.3.5 Experimental Considerations

Finally, interference and appropriate controls are essential considerations during in vitro evaluation for a nanomedicine. Selecting assays to minimize risk of interference is recommended and inhibition/enhancement controls (spike recoveries with the nanomedicine^168^) and nanoparticle only controls should be included on top of positive and negative controls. For example, endotoxin contamination assessment requires controls that evaluate whether nanoparticles themselves can inhibit or enhance the result of the LAL assay.^169^ Results from LAL assays are invalid without inclusion of these controls. In vitro immunotoxicity assessment has similar requirements: The large, charged surface area of nanomaterials can readily interferer with the constituents of the ELISA assay, while many nanomaterials have high absorbance at wavelengths used to measure these assays.^170^ Where possible, nanorelevant positive controls are also recommended. For example, Doxil is known for inducing CARPA,^171^ and silica nanoparticles readily induce inflammasome activation.^172^ These are good positive controls for assessing CARPA and the inflammatory response, respectively.^173^

### 3.4 In Vitro–In Vivo Correlation

In vitro assessment may not always replicate what will occur in vivo. Despite the variety of available immunotoxicity assays, gaps exist between in vitro and in vivo evaluation with nanomedicines and accurately predicting what occurs in vivo based on in vitro data is not always possible. Dobrovolskaia and McNeil evaluated the gaps existing between in vitro and in vivo immunotoxic testing of nanoparticles (**Table** **6**).[qv: 6a] Selecting in vitro tests with efficient predictability and correlation to the chosen in vivo model was the major point brought forward by the authors. Although in vitro studies may not be 100% effective at predicting what occurs in vivo, selecting multiple assays in these cases may give a better prediction for outcome. Careful selection and a knowledge of which assays correlate best for a particular nanomedicine will bridge the gap between in vitro and in vivo.

**Table 6:** Summary of results from in vitro–in vivo immunotoxicity correlation. Summarized from a review by Dobrovolskaia and McNeil[qv: 6a]

Good correlation	Fair correlation
Hemolysis	Thrombogenicity
Complement activation	Myelosuppression
MPS uptake	Immunosuppression
Immune stimulation	

### 3.5 In Vivo Immunotoxicity Assessment

Following on from in vitro evaluation, choosing the most fitting animal model for the nanomedicine in question with respect to its propensity for specific immunotoxic effects is key. Research reviewing suitable animal models for nanomedicine immunotoxicity is scarce and choices of models are limited for academic laboratories. Below are guidelines for the in vivo assessment of some immunotoxicities listed previous.

#### 3.5.1 Thrombogenicity

From Section 3.4, we know that some in vitro models can accurately reflect some immunotoxicities in vivo. However, thrombogenicity, for example, has many layers of regulation (plasma, platelets, leukocytes, and endothelial cells^10^, ^13^) which makes in vitro assessment difficult. Therefore, an efficient animal model is necessary. Mice and rats are currently the most employed animal model for evaluating this immunotoxicity; however, advances are required to improve its correlation. In 2008, Siller‐Matula et al. compared rats, rabbits, pigs, and sheep coagulation profile against humans. Sheep were discovered to have the most similar coagulation time to that of humans, our clot firmness was most comparable to rabbits and maximum lysis values were most related to pigs.^174^ It was therefore concluded that sheep may be the most comparable preclinical model to humans. Rabbits were noted as useful models for platelet interactions and pigs are an effective model for studying fibrinolysis. Therefore, if the specific mechanism of thrombogenicity with a nanomedicine is established, a more correlating animal model can be identified.

#### 3.5.2 Complement Activation

CARPA evaluation has made huge advancements with regards to pigs^54^, ^175^ and recently miniature pig^176^ models. Studies by Szebeni et al.[qv: 175b,177] compared dogs, rats, and pigs as models for severe, life‐threatening human hypersensitivity reactions after infusion with lipid‐based and polymeric nanoparticles. Pigs were noted as having the most similar hypersensitive responses to that of humans and are considered the gold standard for these nanoparticles, exhibiting high reproducibility and sensitivity to low doses of these formulations. It remains to be seen, however, if this model is as relevant toward inorganic cancer nanomedicines.^178^

#### 3.5.3 Immune Stimulation

To detect immune suppression or stimulation in vivo, mice are the most used model (Tables 3 and 4). The lymph node proliferation assay has been suggested as a good model for detecting delayed‐type hypersensitivity effects with nanoparticles below 100 nm, as they can sufficiently evade the MPS and migrate to the draining lymph node after subcutaneous injection. In this procedure, mice are injected subcutaneously (between the ears) with phosphate buffered saline (PBS) (negative control), streptozotocin (positive control) or nanoparticles followed by intravenous administration with ^3^H‐thymidine. After being euthanized, the lymphocytes within the draining lymph nodes are isolated and cultured in vitro to establish their proliferation rate.^5^, ^179^ Of course, subcutaneous injection may not be truly reflective of an intravenously delivered cancer nanomedicine but can provide an outlook on the in vivo effects between a given nanomedicine and lymphocytes. In the literature, however, determining cytokine levels—IL‐6, IL‐1, IL‐8, and TNF‐α, in particular (from Tables 3 and 4)—is the most common method for evaluating immune stimulation with nanoparticles in vivo.

#### 3.5.4 Immune Suppression

Evaluating immunosuppression using bronchial alveolar lavage (BAL) fluid in mice after pulmonary accumulation of nanoparticles has also been used extensively in the literature. In this case, mice are pre‐exposed to an allergen, such as ovalbumin, which induces a Th‐2 immune response. Immune suppression is measured through changes in levels of inflammatory cells in BAL fluid after drug administration.^132^, ^180^ Alternatively, the in vivo T cell‐dependent antibody response assay has recently shown correlation with the in vitro human leukocyte activation assay using the approved iron oxide nanoparticle Feraheme. This assay treats CD‐1 mice with PBS (negative control), cyclosporin (positive immunosuppressive control), Keyhole Limpet Hemocyanin – KLH (known antigen), and nanoparticles. ELISA kits specific for anti‐KLH IgM and anti‐KLH IgG are then used to monitor immunosuppression at various time points after treatment.^181^ More work is necessary to divulge whether this correlation is maintained with different organic and inorganic nanoparticles.

At each stage of immunotoxicity assessment, assay interference and relevance of assay/animal model must be considered to ensure results suffice. Decisions on whether to progress to the next stage of preclinical assessment can then be made.

## 4 Regulatory Considerations

A recent review by Giannakou et al. recalls how immunotoxic effects are regulated with nanomaterials.^182^ This review highlights common framework for immunotoxicity testing based on the International Conference on Harmonization S8 (ICH S8)^183^ guidelines, and gaps currently existing within standard testing. ICH S8 guidelines are in place for pharmaceuticals that are likely to interact with the immune system. As noted throughout, cancer nanomedicines administered intravenously will interact with the immune system, and have the potential to cause an array of unintentional immunotoxic effects. ICH S8 lacks guidelines for CARPA, hypersensitivity, inflammasome activation and myelosuppression. As described previously, some of these immunotoxic effects are extensively reported with nanomedicines used to treat cancer, and so there is a need for standardized approaches. Currently, there are no regulatory‐approved guidelines for evaluating immunotoxicity specifically for nanomedicines. Consequently, this review recommends International Organization for Standardization guidelines (ISO/TS:10993‐20:2006^184^) which are a standardized framework for the detection of immunotoxicities with medical devices. Alternatively, the US NCL^185^ is also recommended. Their work on immunotoxicity with nanomaterials is referenced throughout this review and has been instrumental in advancing this area. They have developed new assays, tested an extensive array of nanomaterials and published many helpful reviews and books in this space. Based on their work, a similar EUNCL has now been established.^186^ Both of these organizations work alongside the FDA and EMA to ensure efficient preclinical evaluation of cancer nanomedicines, with a particular focus on immunotoxicity.

## 5 Future Perspective for Cancer Nanomedicines

### 5.1 Overcoming Immunotoxic Effects in the Future

The next generation of cancer nanomedicines should have a higher success rate in the clinic. Standardized immunotoxicity assessments with endotoxin‐free nanomedicines and appropriate controls for interference will allow for better predictions on where immunotoxicities are likely to emerge, and significantly improve the reliability of literature in this space. In the short term, however, we can learn from the issues and successes of clinically approved nanomedicines and make design considerations and immunotoxicity evaluations based on these cases. Of course, there will always be an element of toxicity when treating cancer. Therefore, in most cases, damage limitation is necessary to alleviate immunotoxic effects. Slower/reduced infusion rates and the co‐administration of antihistamines/ corticosteroids has been adopted by a number of nanomedicines to counteract adverse reactions in the clinic.[qv: 49b,175b,177c,187] Furthermore, alternative modes of delivery have been utilized: Nanotherm therapy relies on intratumoral injection to employ magnetic hyperthermia in the clinic,^188^ Sienna+ uses subcutaneous injection for sentinel lymph node localization,^189^ and there are numerous studies optimizing magnetic delivery of nanoparticles to tumors.^190^ In the future, advances in nanomedicine design (cores, coatings, and APIs), and the development of prognostic tests to stratify patients most likely to respond will overcome the hurdles associated with immunotoxicity and improve clinical translation.

### 5.2 Realistic Expectations

Cost and availability are major issues to consider from an academic perspective when it comes to evaluating immunotoxicity. Endotoxin assessment, for example, is quite an expensive assay for academic laboratories, especially if comparisons between two assays are expected. It is likely that endotoxin levels will be established early using one assay in academic laboratories, with the hope of a full endotoxin assessment when the nanomedicine shows desirable efficacy. A greater knowledge of endotoxin contamination, along with the use of starting materials and environments that are endotoxin‐free should ensure endotoxin becomes less of an issue in the future. Likewise, current in vitro–in vivo correlation and nanomedicine assay interference means that more effective in vitro immunotoxicity assays are necessary to accurately assess a particular immunological effect, increasing the cost of early immunological assessment. Similarly, choosing a fitting animal model for a particular immunotoxicity is not particularly realistic at the academic level either, as laboratories may only have access to what animals are being bred at their local institutional animal facility. Therefore, in order to advance immunotoxicity assessment at the academic level, improving the power of in vitro and ex vivo immunotoxicity assays to reflect what occurs in vivo is key for the future.

### 5.3 Using Immunotoxic Effects for Our Benefit

It is important to note that although only unintentional immunological effects of nanomedicines are discussed here, these same effects have been utilized for many oncological applications. Nanoparticles have shown improved delivery of cytokines,^191^ nucleic acids,^192^ peptides,^193^ and antibodies^194^ to stimulate the immune system into an effective antitumor immune response. Nanomedicines in these cases offer a flexible platform that can be tailored to favor a number of immunological interactions depending on their characteristics. For example, particles closer to the µm range (0.5–2 µm) have been shown to favor dendritic cell (DC) uptake close to the injection site, whereas, nanoparticles in the nanometer range (20–200 nm) can freely drain into lymph nodes and become internalized by DC in that location,[qv: 179a] enhancing adaptive immunity. Moreover, modifying a nanoparticles surface can selectively induce or evade phagocytic uptake,^195^ delivering the nanoparticles to a particular site of the RES or increasing its circulation time respectively. Pairing these characteristics with an inherently immunostimulatory nanomedicine creates an ideal platform for a cancer vaccine. Polypropylene sulfide nanoparticles of a particular size and surface chemistry were shown to controllably activate complement and preferentially target lymph node‐residing DC, subsequently inducing DC activation and orchestrating an adaptive immune response.^196^ This lymph node‐specific targeting was recently employed by Zhu et al. where nucleic acid nanoparticles were associated to tumor neoantigens for simultaneous delivery of adjuvants and antigens to synergistically activate antigen presenting cells and elicit a potent antitumor immune response.^197^ The most successful of these nanovaccines has been the biodegradable polymeric nanoparticles such as poly(lactic‐*co*‐glycolic acid) (PLGA). These nanoparticles have been shown improve antigen uptake into APCs, prevent antigen degradation, and accommodating its timely release for a longer lasting immune response with less administrations required.^198^ Notably, Rietscher et al. encapsulated ovalbumin in a polymer composed of PLGA, PEG, and poly(allyl glycidyl ether) and tested its efficacy in vitro and in vivo.^199^ It was found that these nanoparticles successfully induced ovalbumin‐specific activation of CD8^+^ T cells, resulting in a strong cytotoxic response that suggests these nanoparticles may have an inherent adjuvant effect. It is expected that multifunctional properties of nanovaccines will certainly have a bright future in the treatment of cancer.

## 6 Conclusion

The potential of cancer nanomedicine is unquestionable. It is providing ways to treat and diagnose cancer never achieved before. Particular characteristics of nanoparticles subject them to an array of immunotoxicities that need to be limited, circumvented or prevented to ensure greater success in the future. Research relating to endotoxin contamination assessment, in vitro and in vivo immunotoxicity, and regulation is advancing this area. In the future, this will reduce the number of failures and ensure greater numbers reaching the clinic.

## Conflict of Interest

The authors declare no conflict of interest.
